# Biological manganese-dependent sulfide oxidation impacts elemental gradients in redox-stratified systems: indications from the Black Sea water column

**DOI:** 10.1038/s41396-022-01200-3

**Published:** 2022-02-05

**Authors:** J. V. Henkel, H. N. Schulz-Vogt, O. Dellwig, F. Pollehne, T. Schott, C. Meeske, S. Beier, K. Jürgens

**Affiliations:** Leibniz Institute for Baltic Sea Research Warnemünde, Seestrasse 15, Rostock, 18119 Germany

**Keywords:** Water microbiology, Biogeochemistry

## Abstract

The reduction of manganese oxide with sulfide in aquatic redox-stratified systems was previously considered to be mainly chemical, but recent isolation of the Black Sea isolate *Candidatus* Sulfurimonas marisnigri strain SoZ1 suggests an important role for biological catalyzation. Here we provide evidence from laboratory experiments, field data, and modeling that the latter process has a strong impact on redox zonation in the Black Sea. High relative abundances of *Sulfurimonas* spp. across the redoxcline in the central western gyre of the Black Sea coincided with the high-level expression of both the sulfide:quinone oxidoreductase gene (*sqr*, up to 93% expressed by *Sulfurimonas* spp.) and other sulfur oxidation genes. The cell-specific rate of manganese-coupled sulfide oxidation by *Ca*. S. marisnigri SoZ1 determined experimentally was combined with the in situ abundance of *Sulfurimonas* spp. in a one-dimensional numerical model to calculate the vertical sulfide distribution. Abiotic sulfide oxidation was too slow to counterbalance the sulfide flux from euxinic water. We conclude that microbially catalyzed Mn-dependent sulfide oxidation influences the element cycles of Mn, S, C, and N and therefore the prevalence of other functional groups of prokaryotes (e.g., anammox bacteria) in a sulfide-free, anoxic redox zone.

## Introduction

In productive aquatic systems, the remineralization of organic matter regularly leads to oxygen (O_2_) deficiency and finally to anoxia or even euxinia. This transition is accompanied by the establishment of a pelagic redoxcline that separates the oxic surface from reducing bottom waters. Meromictic lakes are especially prone to such developments but semi-restricted water bodies, such as fjords and brackish/marine and hypersaline basins, are vulnerable as well [1–5]. The Black Sea is the world’s largest semi-enclosed basin and the type-locality for density-stratified aquatic systems, as severe bottom water euxinia is prevalent since ~8000 years [6, 7]. In the Black Sea redoxcline, a layer in which O_2_ and sulfide (S^2−^, here defined as the sum of H_2_S, HS^−^, and S^2−^) are virtually absent, referred to as the “suboxic zone,” has been frequently reported since 1989 [1, 6, 8–11]. This zone may span tens of meters but it is not well-defined, as some descriptions also encompass waters with low concentrations of O_2_ (<5 µM [8]; <3 µM [12]; <1 µM [13]) and S^2−^ (<0.2 µM [12]; <0.1 µM [13]). In the following, we refer to the zone of transition from oxic to anoxic and euxinic water conditions as the redoxcline.

Due to the separation of O_2_ and often nitrate (NO_3_^−^) and nitrite (NO_2_^−^) from S^2−^ in the redoxcline of the Black Sea, the depletion of S^2−^ at the upper boundary of euxinic water (also known as chemocline) has been attributed to lateral intrusions of oxygenated waters [12, 14] or to the intense cycling of the redox-sensitive trace metal manganese (Mn) [10, 15]. In their one-dimensional biogeochemical model, Yakushev et al. [16] reproduced the vertical geochemical water column profile measured in the Black Sea without lateral intrusion, instead identifying reduced and oxidized Mn species as the main drivers of O_2_ reduction and S^2−^ oxidation in the redoxcline. Although lateral intrusions from the Bosporus plume may affect the redoxcline in the far southwestern part of the Black Sea [14], extensive data gathered over a two-year period by Stanev et al. using Argo floats [11] support this one-dimensional model and the importance of a manganese shuttle.

During Mn-cycling, dissolved Mn^2+^ and intermediate Mn^3+^ are transported upwards by diffusion [1, 12, 15, 17, 18], and thus from reducing towards oxygenated waters, where both Mn species are then oxidized [19]. The resulting Mn (IV) oxide particles (here referred to as MnO_2_) sink back towards the underlying euxinic water [20], where they are reduced to dissolved Mn^3+^ and Mn^2+^ by S^2−^ and/or Fe^2+^ [21]. The chemical oxidation of S^2−^ by MnO_2_ in laboratory experiments was shown to occur rapidly (within minutes) [22, 23]. However, in those studies, MnO_2_ was supplied in millimolar concentrations, i.e., several magnitudes larger than the natural levels in the Black Sea [1, 9, 10]. Because the chemical oxidation of S^2−^ by MnO_2_ follows second-order reaction kinetics, such that the concentrations of both reactants affect the overall speed of the reaction [23], the laboratory results on chemical oxidation may not represent the in situ processes in the Black Sea.

The recent description of S^2−^ oxidation coupled to the reduction of MnO_2_ by the Black Sea isolate *Candidatus* Sulfurimonas marisnigri SoZ1 (phylum *Campylobacterota*, class *Campylobacteria* [24]), isolated from the upper boundary of euxinic water, supports a biological mode of S^2−^ oxidation in the absence of O_2_ and NO_3_^−^ [25]. In laboratory experiments, *Ca*. S. marisnigri SoZ1 oxidized S^2−^, elemental sulfur (S^0^), and thiosulfate (S_2_O_3_^2−^) to sulfate (SO_4_^2−^), with MnO_2_ as the sole electron acceptor [25, 26]. The reduction of MnO_2_ proceeded by its conversion to Mn^3+^ and further to Mn^2+^, which precipitated as Ca-rich Mn-carbonate [25]. A biological mode of S^2−^ oxidation with MnO_2_ may therefore account for the observed accumulation of the intermediate Mn^3+^ in the redoxcline [1, 12], for the chemosynthetic production of *Campylobacterota* in the absence of O_2_ and NO_3_^−^ [27], and for the maintenance of anoxic and non-sulfidic zones in the redoxcline. However, in the absence of data on the abundance, activity, and taxonomy of putative S-oxidizing and Mn-reducing bacteria, the impact of Mn-dependent S oxidation on the water column geochemistry of the Black Sea is unclear and unquantified.

Our study investigated the potential contribution of extent microbial S^2−^ oxidation by *Sulfurimonas* spp., using MnO_2_ as terminal electron acceptor, to the overall S^2−^ oxidation in the redoxcline of the Black Sea. For this purpose, cell-specific S^2−^ oxidation rates with MnO_2_ by the representative isolate *Ca*. S. marisnigri SoZ1 (=JCM 39139; =DSM 111879) were determined in laboratory experiments together with assessments of the in situ abundances and gene expression of *Sulfurimonas* spp. across the redoxcline of the Black Sea. The cell-specific oxidation rates and the in situ abundance were then combined to model the S^2−^-concentration profile in the Black Sea. The results provide strong evidence that the activity of *Sulfurimonas* spp. contributes significantly to anaerobic sulfide oxidation and to the formation of a sulfide-free, anoxic zone, both in the Black Sea and potentially in other redox-stratified systems.

## Material and methods

The materials and methods are described here in brief. A detailed description of the Material and methods can be found in the supplementary information.

### Field sampling

**Fig. 1:**
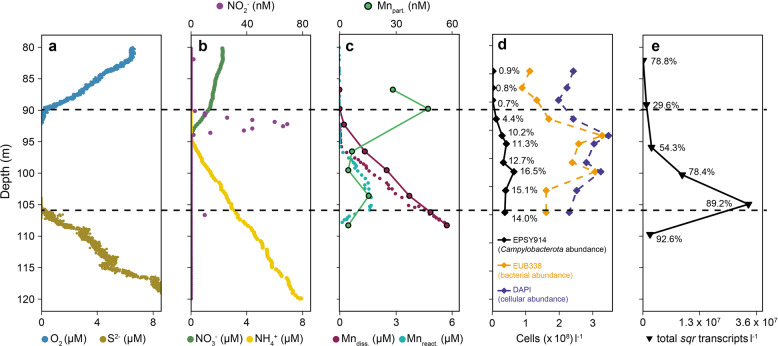
Vertical water-column profile of the Black Sea redoxcline at station 32. Horizontal dashed lines indicate the approximate boundaries of the redoxcline. **a** Oxygen (O_2_) and sulfide (S^2−^). **b** nitrate (NO_3_^−^), nitrite (NO_2_^−^), and ammonia (NH_4_^+^). **c** particulate manganese (Mn_part._), total dissolved manganese (Mn_diss._), and dissolved reactive manganese (Mn_react_ ≃ Mn^3+^ [1, 12, 18]). **d** Cellular abundance of cells positively stained with CARD-FISH probes EPSY914 (*Campylobacterota*) and EUB338 (*Bacteria*) and with the DNA dye DAPI (total cells). The relative abundance of EPSY914-positive cells vs. total DAPI-stained cells is expressed as a percent. **e** Total transcript abundance of the sulfide:quinone oxidoreductase gene (*sqr*). The relative abundance of *sqr* transcripts expressed by *Sulfurimonas* spp. is expressed as a percent. The data shown in **a** and **b** are from Schulz-Vogt et al. [9]. In **c**, the larger black-enclosed circles and solid lines represent data from bottle casts at station 32; the small non-enclosed circles represent data from high-resolution pump CTD profiles taken at station 66 and aligned according to density.

Samples were taken during a cruise with the R/V “Maria S. Merian” (MSM33) in November/December 2013 and sampling was performed as described previously [9]. All data presented herein originated from station 32, located in the western central gyre of the Black Sea (43° 31.922′ N, 32° 30.909′ E; water depth 2070 m), with the exception of the high-resolution data on total dissolved Mn (Mn_diss_) and dissolved reactive Mn (dMn_react_; [18]), obtained from station 66 (43° 31.8019′ N, 36° 05.9960′ E; water depth 2177 m). The characteristics of the water columns of stations 32 and 66 were similar, as indicated by the disappearances of O_2_ at densities (σ_θ_) of 15.93 and 15.94 and of S^2−^ at *σ*_θ_ of 16.15 and 16.16, respectively. The profiles of O_2_ and S^2−^ at both stations were vertically separated by approx. 15 m thick layer (shown in Fig. 1a for station 32). Water column profiles from successive casts were constructed by aligning the data according to *σ*_θ_ of cast P0014F13 (station 32), as done previously [9].

### Gases, nutrients, and metals

Data for the water column profiles of dissolved gases (O_2_, H_2_S) and nutrients (NO_3_^−^, NO_2_^−^, NH_4_^+^) were taken from Schulz-Vogt et al. [9]. The concentrations of Mn_diss_ and dMn_react_ as well as those of particulate Mn (Mn_part_) were measured by inductively coupled plasma optical emission spectrometry, according to the protocols reported in [18] and [10].

### Catalyzed reporter deposition fluorescence in situ hybridization (CARD-FISH)

CARD-FISH was carried out following the protocols of Pernthaler et al. [28] and Sekar et al. [29], modified as described in [30]. For the enumeration of *Bacteria*, a mixture of horseradish-peroxidase-labeled oligonucleotide probes (EUB338, EUB338-II, EUB338-III [31]) was used. *Campylobacterota* were enumerated using probe EPSY914 [27]. Non-specific binding was determined using the NonEUB probe [32]. Filter sections were counterstained with DAPI (1 mg ml^−1^) and inspected using an epifluorescence microscope (Axioscope, Carl Zeiss) together with filter sets 01 (DAPI) and 10 (Alexa 488).

### Sampling, processing, and data analysis of metagenomic and metatranscriptomic data

#### Amplicon sequencing of the 16S rRNA gene and 16S rRNA

Samples for 16S rRNA gene and 16S rRNA amplicon sequencing were extracted using the AllPrep DNA/RNA kit (Qiagen; Hilden, Germany). DNA extracts were stored directly; from the extracted RNA, a maximum of 100 ng was DNase-treated using the Turbo DNA-free kit (Thermo Fisher Scientific; Waltham, MA, USA). The DNase-treated RNA (max. 20 ng) was reverse transcribed using Multiscribe RT (Thermo Fisher Scientific). All samples of DNA and treated RNA, including additional Mock Community samples from Zymo Research (Freiburg, Germany) as controls, were sent to LGC Genomics (Berlin, Germany) for sequencing with MiSeq (Illumina). The resulting sequences were analyzed using the SILVA_NGS pipeline (release 138.1) [33, 34], with settings and OTU clustering based on 97% similarity, as described previously [35].

### Metagenomic and metatranscriptomic analyses

Sampling and processing were done as described [9]. Genes annotated as *Campylobacterota* were extracted and sulfur oxidation genes were identified based on functional annotation, sequence similarity, and gene synteny (detailed description in the supplementary information). The identified genes were taxonomically annotated using CAT (v5.2.3), based on the Diamond aligner (v2.0.8.147 [36]) in blastp mode against the NCBI’s NR database (as of July 1, 2021), with the r-parameter set to 3 [37].

### Estimation of the rates of S^2−^ oxidation with MnO_2_ by *Ca*. S. marisnigri SoZ1

The rates of biological S^2−^ oxidation with MnO_2_ by the isolate *Ca*. S. marisnigri SoZ1 were determined by spiking anoxic, constantly stirred medium containing MnO_2_ and *Ca*. S. marisnigri SoZ1 or no bacteria either five (sterile controls) or eight (with *Ca*. S. marisnigri SoZ1) times with Na_2_S to obtain S^2−^ concentrations of 20–30 µM. The decline in the S^2−^ concentration was documented in three replicates for each treatment using an H_2_S and pH microsensor (Unisense, Aarhus, Denmark). The reactions in the replicates were then inhibited either by the addition of sodium azide (not shown) or by pasteurization, followed by another three additions of Na_2_S. The H_2_S concentrations measured in the experiment were corrected for temperature, salinity, and pH to obtain the S^2−^ values.

The overall reaction can be described by Eq. 1, which can be transposed to Eq. 2 by normalizing the S^2−^ concentration at time t to the S^2−^ concentration at time t_0_.

1 $$\left[ {{{{{\rm{S}}}}}^{2 - }} \right]_t = \left[ {{{{{\rm{S}}}}}^{2 - }} \right]_0e^{\left( {a + kt} \right)}$$

2 $$\frac{{\left[ {{{{{\rm{S}}}}}^{2 - }} \right]_t}}{{\left[ {{{{{\rm{S}}}}}^{2 - }} \right]_0}} = e^{\left( {a + kt} \right)}$$

where [S^2−^]_t_ is the concentration of S^2−^ at time t; [S^2−^]_0_ is the initial concentration of S^2−^, a as a correction variable for the y-intercept; *k* is the reaction rate coefficient (s^−1^); and t is the time in seconds.

The non-linear least-squares fit of Eq. 2 was performed with R (version 3.5.1) for individual spikes of Na_2_S to determine the overall reaction rate coefficient *k*. The biological reaction rate (*k*_bio_) was calculated by subtracting the value of *k* after pasteurization from the overall reaction rate coefficient *k* before pasteurization. The value of *k*_bio_ was divided by the cellular abundance of *Ca*. S. marisnigri SoZ1 to obtain a cell-specific reaction rate coefficient (*k*_cell_), with the mean value (*k*_cell_ = −1.05 × 10^−13^ l cell^−1^ s^−1^) used in subsequent numerical modeling.

### Modeling the S^2−^ concentration profile of the Black Sea

The impact of S^2−^ oxidation activity by *Sulfurimonas* spp. on the geochemical water column profile in the Black Sea was estimated by combining data on the abundance of *Sulfurimonas* spp. in the Black Sea and the cell-specific S^2−^ oxidation rates of *Ca*. S. marisnigri SoZ1 in a numerical model. In the modeling approach of [38], the differential equation for diffusive transport is described by Eq. 3:

3 $$\delta c/\delta t = D\delta ^2c/\delta x^2$$

where *D* is the diffusion coefficient; c is the concentration; *t* is the time; and *x* is the distance-coordinate. The equation is solved using the explicit numerical solution obtained with Eq. 4, adapted from [9]:

4 $$C_{S^{2 - }(x,t + \Delta t)} = {C_{S^{2 - }\left( {x,t} \right)} + \frac{{\Delta t \times D_x \times \left( {C_{S^{2 - }\left( {x + \Delta x,t} \right)} - 2 \times C_{S^{2 - }\left( {x,t} \right)} + C_{S^{2 - }\left( {x - \Delta x,t} \right)}} \right)}}{{\Delta x^2}} + }$$

4.1 $$\Delta t \times k_{{{{{\rm{cell}}}}}} \times CA_{{{{{\rm{EPSY914}}}}}\left( x \right)} \times C_{S^{2 - }\left( {x,t} \right)} \times F_{{{{{\rm{Sulfurimonas}}}}}\,{{{{\rm{in}}}}}\,{{{{\rm{Campylobacterota}}}}}}$$

4.2 $$\Delta t \times k_{{{{{\rm{chem}}}}}} \times C_{S^{2 - }(x,t)}$$

where C_S_^2−^ is the concentration of S^2−^ at a given water depth *x* and time *t*, *D*_x_ is the diapycnal diffusivity at water depth *x*, as determined in [17] (we set 4 × 10^−6^ or 1 × 10^−6^ m^2^ s^−1^ as the upper and lower limits of diapycnal diffusivity). The consumption of S^2−^ during biological or chemical oxidation was taken into account in Eq. 4, by adding either Eq. 4.1 or Eq. 4.2, respectively. The rate of biological S^2−^ oxidation was calculated by multiplying the cell-specific reaction rate coefficient of *Ca*. S. marisnigri SoZ1 (*k*_cell_ = −1.05 × 10^−13^ l cell^−1^ s^−1^) by the cellular abundance of *Sulfurimonas* spp. and the local S^2−^ concentration. The chemical S^2−^ oxidation rate was calculated using a chemical reaction rate coefficient (*k*_chem_ = −9.53 × 10^−8^ s^−1^ [23] based on a constant MnO_2_ concentration of 10 nM, pH 7, and 10 °C) and the local S^2−^ concentration. The starting condition of the model was 10 µM S^2−^ at 120-m water depth. Oxidation at the upper boundary of euxinic water (106 m water depth) and above was assumed based on the vertical concentration profile of S^2−^ (see the “Discussion” for details). The Excel-based spreadsheet of the model is provided in the Supplementary Material.

### Growth of *Ca*. S. marisnigri SoZ1 with S^2−^ and MnO_2_ in semi-continuous culture

The semi-continuous culture experiment was performed by [25] to identify the reaction end-products of MnO_2_ and S^2−^ under sterile conditions and in the presence of *Ca*. S. marisnigri SoZ1. The method is described in the supplement. In the experiment, the cellular abundance of *Ca*. S. marisnigri SoZ1 was proportional to the applied S^2−^ flux, and the S^2−^ flux per cell was calculated to allow comparisons with the S^2−^ fluxes in the upper boundary of euxinic water in the Black Sea redoxcline.

## Results

### Geochemical structure of the pelagic redoxcline of the Black Sea

In the central western gyre of the Black Sea, the zone where O_2_ and S^2−^ became undetectable (<0.2 µM O_2_, <1 µM S^2−^) was located roughly between 90 and 105 m water depth (Fig. 1a). Concentrations of O_2_ above and S^2−^ below this zone increased steadily. Analyses conducted using an ultra-low switchable trace-oxygen sensor (STOX) [41], with a detection limit of <10 nM, did not detect O_2_ traces below the NO_2_^−^ peak at 92 m water depth. Thus, at the time of sampling the redoxcline below 92 m was defined as anoxic. The concentration of NO_3_^−^ decreased continuously from 80 m to 90 m water depth, followed by a steeper slope from 90 m, where the O_2_ concentration fell to < 0.5 µM, to 95 m, where NO_3_^−^ was depleted (Fig. 1b). The decrease in NO_3_^−^ between 90 and 95 m water depth coincided with the marked production of NO_2_^−^. Ammonia (NH_4_^+^) decreased linearly from the euxinic water towards 95 m water depth, where it became undetectable (Fig. 1b).

Particulate Mn concentrations were highest (50 nM) in the upper part of the redoxcline, where O_2_ became depleted, with a second peak (16 nM) at ~104 m water depth (Fig. 1c). With the exception of the second peak, the concentration of Mn_part._ generally decreased with increasing water depth. By contrast, Mn_diss._ increased continuously beginning at ~95 m water depth and continuing into euxinic water, reaching 6 µM at 110 m water depth. Since high-resolution data for Mn_diss_ and dMn_react_ were not available for the station in the central western gyre, data from the central eastern gyre (2177 m water depth) were aligned according to density and shown instead. In agreement with Trouwborst et al. [12] and Dellwig et al. [1], Mn_diss._ within the redoxcline was almost entirely composed of the intermediate Mn^3+^, measured in this study indirectly as dMn_react_. [18]. The concentration of dMn_react._ peaked at the upper boundary of euxinic water at ~105 m water depth and then decreased sharply below, that is, in the first few meters of euxinic water.

### Cellular abundance and S^2−^ oxidation activity in the redoxcline of the Black Sea

Total cellular abundance at station 32 increased from ~2 × 10^8^ cells l^−1^ above and below the redoxcline to ~3 × 10^8^ cells l^−1^ in the intervening layer (Fig. 1d). Likewise, total bacterial cell counts determined using the FISH probe EUB338[I-III] were elevated within the redoxcline and accounted for 50 to 89% of the total cell counts, depending on the water depth. The abundance of *Campylobacterota* increased steadily towards the euxinic water, from ~2 × 10^6^ cells l^−1^ in oxic water to ~4 × 10^7^ cells l^−1^ at the upper boundary of euxinic water, with the highest abundance (6.5 × 10^7^ cells l^−1^) occurring at 100 m water depth (Fig. 1d). The relative abundance of *Campylobacterota* (% of DAPI counts) increased as well, from >1% at the upper boundary of the redoxcline at 89 m to ~15% at the upper boundary of euxinic water at 106 m water depth (Fig. 1d).

Transcription of the sulfide:quinone oxidoreductase gene (*sqr*), a key enzyme in the oxidation of S^2−^ to S^0^ and polysulfides [42], was used as a molecular marker of bacterial S^2−^ oxidation across the redoxcline. The expression of *sqr* was detectable at all sampled water depths and increased across the redoxcline by more than two orders of magnitude, from 2.6 × 10^5^ transcripts l^−1^ in oxic water to 3.8 × 10^7^ transcripts l^−1^ at the upper boundary of euxinic water (~105 m), before undergoing a steep decline (by 93%) below (~110 m) (Fig. 1e). Because we chose a conservative method for the taxonomic annotation of *sqr* and other sulfur oxidation genes (CAT with r-parameter set to 3), the taxonomic annotation of these genes was restricted to the family level (*Helicobacteraceae*), but with high confidence. However, since *Sulfurimonas* spp. comprised 99.9–100% of 16S rRNA gene reads related to the family *Helicobacteraceae*, transcription of *sqr* could be assigned exclusively to the genus *Sulfurimonas*. The relative transcription level of *sqr* by *Sulfurimonas* spp. for all detected *sqr* transcripts increased with water depth, from 29.6% at ~86 m to 92.9% at ~110 m. At ~105 m, *Sulfurimonas* spp. expressed 89.2% of the total *sqr* transcripts.

### Sequence-based abundance and the taxonomic structure of the phylum *Campylobacterota*

**Fig. 2:**
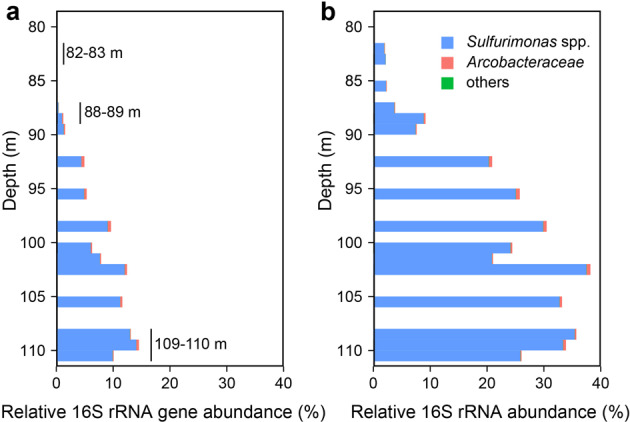
Taxonomic composition of the phylum *Campylobacterota* in the redoxcline of the Black Sea. Relative 16S rRNA gene (**a**) and 16S rRNA (**b**) abundance of the phylum *Campylobacterota* across the redoxcline at station 32 from three density-aligned consecutive CTD casts shown at 1-m resolution. The relative abundances of the 16S rRNA gene and of *Campylobacterota*-related 16S rRNA gene reads increased across the redoxcline towards euxinic bottom waters. Relative abundances of the 16S rRNA gene and of 16S rRNA of *Sulfurimonas* spp. (blue) increased with depth, from < 1% to ~14% and from ~2% to > 35%, respectively (Tables S1 and S2). The relative read abundance of *Sulfurimonas* spp. within *Campylobacterota* increased from ~70% to ~99% across the redoxcline, based on 16S rRNA gene abundance; 16S rRNA abundance was always > 95% (Tables S3 and S4). Relative bacterial abundance of the family *Arcobacteraceae* (red) and the remaining *Campylobacterota*-related reads (others, green) were low (*Arcobacteraceae*) or too low to be visible in the figure (others).

Relative 16S rRNA gene and 16S rRNA abundances of *Camplyobacterota* increased steadily across the redoxcline (Fig. 2), in line with the results of microscopic counting using the CARD-FISH probe EPSY914, targeting the phylum *Campylobacterota* (Fig. 1d). The relative abundance of *Campylobacterota*-related 16S rRNA gene and 16S rRNA reads increased over a water depth of ~82–110 m, from ~0.1% to ~ 15% and from ~2% to ~35%, respectively (Fig. 2, Tables S1 and S2). Within *Campylobacterota*-specific reads, those belonging to *Sulfurimonas* spp. dominated, accounting for ~70% (16S rRNA gene) and >95% (16S rRNA) at 82 m water depth and >96% (16S rRNA gene) and >99% (16S rRNA) at the upper boundary of euxinic water (Tables S3 and S4). For *Sulfurimonas* spp., relative 16S rRNA abundance was 2.5 times higher than the corresponding relative abundance of the 16S rRNA gene in the same sample (*R*^2^ = 0.92). According to these findings, *Sulfurimonas* spp. accounted for almost all of the phylum *Campylobacterota* and was metabolically active across the redoxcline.

### Sulfur oxidation activity of *Sulfurimonas* spp. in the Black Sea redoxcline

**Fig. 3:**
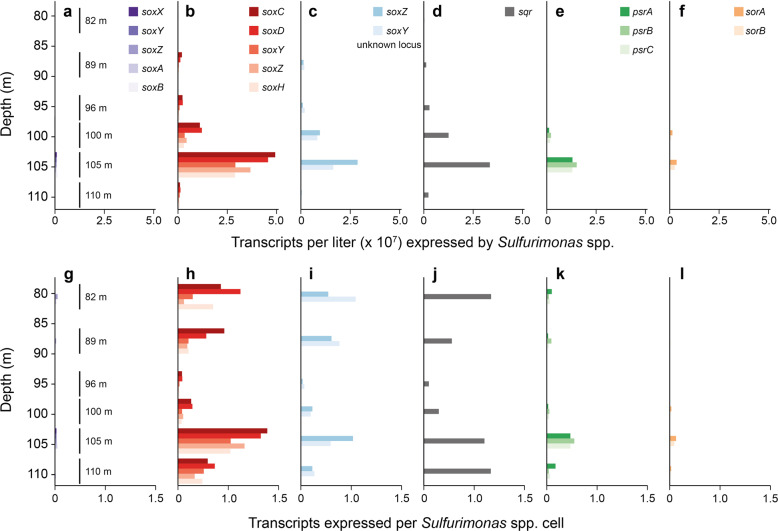
Transcription of the S oxidation genes across the redoxcline with taxonomic annotation to the genus *Sulfurimonas*. **a**–**f** Total number of transcripts expressed by *Sulfurimonas* spp.; **g**–**l** transcript abundance, normalized to the cellular abundance of *Sulfurimonas* spp. determined in *Campylobacterota* by CARD-FISH (probe EPSY914, Fig. 1d), multiplied by the relative 16S rRNA gene abundance of *Sulfurimonas* spp. within *Camplyobacterota*-specific reads (Table S1). Sampled depths are displayed in (**a**) and (**g**).

The analysis of the S oxidation genes expressed by *Sulfurimonas* spp. revealed, besides *sqr*, two sulfur oxidation (sox) clusters (*soxXYZAB* and *soxCDYZH*), polysulfide reductase (*psrABC*), and sulfite:cytochrome c oxidoreductase (*sorAB*) (Fig. 3). In agreement with the water column profile of S^2−^, the expression of these genes was highest at the upper boundary of euxinic water (105 m water depth; Fig. 3a–f). The expression of *soxCDYHZH* was approximately two orders of magnitude higher than that of *soxXYZAB* (Fig. 3a, b). Transcripts of *soxY* and *soxZ* were also detected on small contigs that could not be assigned to cluster *soxXYZAB* or *soxCDYZH*, respectively (uncertain loci, Fig. 3c, i). The transcript abundance and vertical expression pattern of *soxCDYZH* were similar to those of *sqr* (Fig. 3b, d), suggesting that S^2−^ oxidation by *sqr* continued via *soxCDYZH*. The cellular-abundance-related transcription of *Sulfurimonas* spp.-annotated genes was calculated by dividing the respective transcript numbers by the cellular abundance of *Sulfurimonas* spp., determined by multiplying the relative 16S rRNA gene abundance of *Sulfurimonas* by the total cellular abundance of DAPI-stained cells. Cellular-abundance-related (normalized) transcript numbers indicated that *sqr* and *soxCDYZH* were equally expressed also in the upper part of the redoxcline towards oxic water (Fig. 3h, j). The normalized expression patterns of *psrABC* (Fig. 3k) and *sorAB* (Fig. 3l) did not resemble or were far less obvious than those of *sqr* and *soxCDYZH*, suggesting that *psrABC* and *sorAB* activities were not directly coupled to the activities of *sqr* and *soxCDYZH*.

### Rates of manganese-oxide-mediated sulfide oxidation by *Ca*. S. marisnigri SoZ1 in lab experiments

**Fig. 4:**
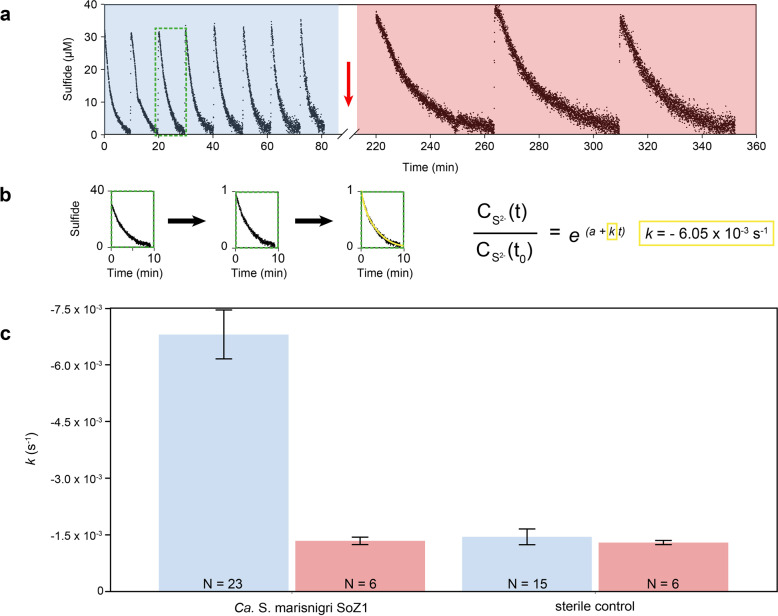
Rates of MnO_2_-mediated sulfide oxidation by *Ca*. S. marisnigri SoZ1. **a** Consumption of S^2−^ in medium containing MnO_2_ and *Ca*. S. marisnigri SoZ1, was recorded using H_2_S microsensors. After pasteurization of the samples (red arrow), S^2−^ removal was considerably slower. **b** Calculation of the reaction rate coefficient *k* (s^−1^) in response to individual S^2−^ spikes and based on the normalized S^2−^ concentrations with a non-linear least-squares fit using the displayed equation. **c** Reaction rate coefficients for the biological treatment with *Ca*. S. marisnigri SoZ1 and the sterile control, before (blue) and after (red) sample pasteurization (mean ± SD). N refers to the total number of Na_2_S spikes as recorded in three replicates for each biological treatment and sterile control.

The slope and linear shape of the vertical concentration profile of S^2−^ (Fig. 1a) indicated high S^2−^ oxidation rates at the upper boundary of euxinic water (~105 m) in the absence of O_2_ and NO_3_^−^ (Fig. 1a, b) but in the presence of MnO_2_ as an available electron acceptor for S^2−^ oxidation (Fig. 1c). The potential impact of biological S^2−^ oxidation on total S^2−^ oxidation was quantified in a lab experiment in which the rate of S^2−^ oxidation by *Ca*. S. marisnigri SoZ1 using MnO_2_ as the electron acceptor was determined (Fig. 4). In biological treatments containing ~5 × 10^7^ *Ca*. S. marisnigri SoZ1 cells ml^−1^, ~30 µM S^2−^ was removed within 10 min, compared to ~45 min in the abiotic controls (Fig. 4a). After thermal inhibition by pasteurization (Fig. 4a, red arrow), the speed of S^2−^ removal in the biological treatments was indistinguishable from that in the abiotic treatments. The reaction rate coefficient *k* for the consumption of each S^2−^ spike was calculated individually (Fig. 4b) and showed that pasteurization lowered the reaction rate to the chemical background level (Fig. 4c). The biological reaction rate coefficient (*k*_bio_) was calculated by subtracting the S^2−^ removal rate after pasteurization from the overall rate before pasteurization; dividing the result by the cellular abundance of *Ca*. S. marisnigri SoZ1 in the respective replicate yielded the cell-specific kinetic rate coefficient (*k*_cell_). The mean value of *k*_cell_ (−1.05 × 10^−13^ l cell^−1^ s^−1^) was used in downstream numerical modeling. To illustrate, with 9 × 10^5^ *Sulfurimonas* spp. cells l^−1^, the biological S^2−^ oxidation rate in the model would be equal to that of chemical oxidation (*k*_chem_), which is described below.

### Modeling sulfide oxidation in the Black Sea redoxcline

**Fig. 5:**
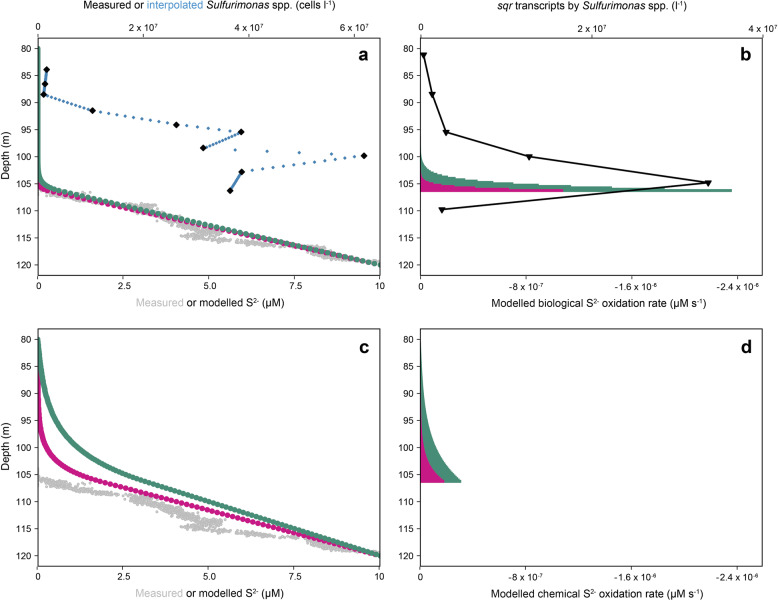
Modeling the S^2−^ concentration profile across the redoxcline of the Black Sea. Results of the modeling of the S^2−^ concentration profile in the Black Sea based on biological S^2−^ oxidation catalyzed by *Sulfurimonas* spp. **a**, **b** or chemical oxidation with MnO_2_ (**c**, **d**) with a diapycnal diffusivity coefficient D_x_ of either 1 × 10^−6^ m^2^ s^−1^ (violet) or 4 × 10^−6^ m^2^ s^−1^ (dark green). The in situ S^2−^ concentration profile (gray dots) was taken from Schulz-Vogt et al. [9], using data originating from the same sampling campaign and same station. Modeled data of the S^2−^ concentration (**a**, **c**) and S^2−^ oxidation rate (**b**, **d**) are shown. The abundance of *Sulfurimonas* spp. (black diamonds) and the interpolated abundance (blue) in the biological model were calculated as the product of EPSY914-positive cell counts and the relative 16S rRNA gene abundance of *Sulfurimonas* spp. within the total reads annotated as *Campylobacterota*.

The potential impact of S^2−^ oxidation by *Sulfurimonas* spp. on the vertical concentration profile of S^2−^ (Fig. 1a) was estimated by assuming either biological or chemical S^2−^ oxidation on the diffusive supply of S^2−^ from euxinic water at the upper boundary of those waters and above (Fig. 5). For the biological model, the cell-specific S^2−^ oxidation rate kinetics (*k*_cell_) estimated with *Ca*. S. marisnigri SoZ1 (Fig. 4) were combined with the natural abundance of *Sulfurimonas* spp. (Fig. 5a). Chemical oxidation was considered as constant, with *k*_chem_ = −9.53 × 10^−8^ s^−1^ (calculated after [23] with 10 nM MnO_2_, pH 7, and 10 °C).

A steady state was reached in both the chemical and the biological models, but the resulting S^2−^ concentration profiles differed (Fig. 5a, c). In the biological model (Fig. 5a, b), 95% of the modeled S^2−^ oxidation activity across the redoxcline occurred over a narrow depth horizon, between ~105 m and ~106 m (*D*_x_ = 1 × 10^−6^ m^2^ s^−1^; Fig. 5b, violet) or ~103 m and ~106 m (*D*_x_ = 4 × 10^−6^ m^2^ s^−1^; Fig. 5b, dark green) water depth, due to the in situ distribution of *Sulfurimonas*. This led to the almost linear shape of the modeled S^2−^ concentration profile (Fig. 5a), which was similar to the observed one (gray dots). The expression of *sqr* by *Sulfurimonas* spp. across the redoxcline was in line with the modeled S^2−^ oxidation rate (Fig. 5b, black triangles). The modeled S^2−^-concentration profile based on a purely abiotic reaction deviated from the measured data (Fig. 5c). In particular, abiotic S^2−^ oxidation rates (Fig. 5d) at the upper boundary of euxinic water were at least one order of magnitude lower than the biological S^2−^ oxidation rates (Fig. 5b) and too low to counterbalance S^2−^ fluxes from euxinic water. In the model based on chemical oxidation, 95% of S^2−^ oxidation across the redoxcline occurred over a broad depth horizon, between ~97 m and ~106 m (*D*_x_ = 1 × 10^−6^ m^2^ s^−1^; Fig. 5d, violet) or ~90 m and 106 m (*D*_x_ = 4 × 10^−6^ m^2^ s^−1^; Fig. 5d, dark green) water depth, which resulted in a large curvature in the S^2−^ profile (Fig. 5c).

## Discussion

### Field-based indications of S oxidation with MnO_2_ catalyzed by *Sulfurimonas* spp. in the Black Sea redoxcline

In line with earlier research, an anoxic zone free of O_2_ and S^2−^ spanning ~15 m was detected within the pelagic redoxcline of the western central gyre (Fig. 1a) [10, 11, 43]. Putative O_2_ contamination was avoided during sampling by measuring O_2_ directly in the outflow of the pump-CTD system using custom-made micro-sensors within glass tubes. O_2_ traces below 92 m water depth could not be detected, even by the ultra-sensitive O_2_ STOX sensors [41] (Fig. 1a). Thus, the redoxcline below 92 m water depth was considered to be completely anoxic. Vertical profiles of dissolved and particulate Mn species indicated the active shuttling of redox equivalents across the redoxcline (Fig. 1c), thereby connecting oxic and euxinic water as previously shown [1, 10, 15, 21]. Based on the S^2−^ concentration profile, S^2−^ oxidation was presumed to proceed at the upper boundary of euxinic water and above, because the linear shape of the profile indicated diffusive transport of S^2−^ towards the redoxcline, with no net production or consumption [44]. Across the redoxcline, Mn_diss._ mainly consisted of Mn^3+^ (Fig. 1c), in agreement with previous results [1, 10, 12]. Whether Mn^3+^ was generated via biological or chemical reduction could not be determined. However, the reduction of MnO_2_ with S_2_O_3_^2−^ by *Ca*. S. marisnigri SoZ1 in laboratory experiments resulted in the accumulation of Mn^3+^ before Mn(II) precipitated as Ca-rich Mn-carbonate [25], suggesting that Mn^3+^ is generated biologically by MnO_2_ reduction with S^2−^. The Ca-rich Mn-carbonate that formed in culture [25] may also have been the source of the often-observed second peak of Mn_part_ occurring at the lower boundary of the redoxcline [10, 45, 46], since Mn^3+/2+^ oxidation to particulate MnO_2_ would have been unlikely given the absence of oxidants.

In line with the model’s results, *sqr* transcription, as an indicator of biological oxidation, peaked at the upper boundary of euxinic water, where the bulk of S^2−^ oxidation activity would be expected, and dropped sharply within euxinic water (Fig. 1e). The sox gene cluster *soxCDYZH* [47–49] was highly expressed as well (Fig. 3b, d), indicating its functional interaction with *sqr*. A previous study reported up-regulation of the *soxCDYZH* cluster under S^2−^ and S^0^ oxidizing conditions [47]. The tetrameric complex Sox(CD)_2_ oxidizes the sulfane sulfur bound to SoxYZ to sulfone sulfur, which is hydrolytically released as SO_4_^2−^ by SoxB [42, 50]. However, *soxB* was hardly expressed (Fig. 3a), such that SoxH in the cluster *soxCDYZH* likely acted as a hydrolase homolog of SoxB, as suggested earlier [47]. The low-level expression of cluster *soxXYZAB* (Fig. 3a) indicated that S_2_O_3_^2−^ was not an important intermediate for *Sulfurimonas* spp. at the upper boundary of euxinic water in the Black Sea water column [47, 51–53]. The expression of *psrABC* indicated S^0^ reduction [54], possibly using the S^0^ formed, for example, by the abiotic oxidation of S^2−^ with MnO_2_ [22, 55, 56] prior to Sqr- and SoxCDYZH-mediated oxidation. In summary, the pattern of gene expression by *Sulfurimonas* spp. was consistent with the complete oxidation of S^2−^ and S-intermediates to SO_4_^2−^ at the upper boundary of euxinic water and in the absence of O_2_, NO_3_^−^, and NO_2_^−^.

### Model-derived indication for S^2−^ oxidation with MnO_2_ catalyzed by *Sulfurimonas* spp. in the Black Sea redoxcline

The cell-specific S^2−^ oxidation rates of *Ca*. S. marisnigri SoZ1 obtained in the lab experiments were fast enough to account for the S^2−^ concentration profile in the Black Sea obtained in a one-dimensional numerical model based on the in situ abundance of *Sulfurimonas* spp. (Fig. 5a, b). By contrast, the chemical oxidation of S^2−^ with MnO_2_ was about one order of magnitude slower (Fig. 5b, d), which resulted in a S^2−^ concentration profile with a large curvature (Fig. 5c). Therefore, S^2−^ oxidation was most likely biological, as also suggested by Mn^3+^ formation (Fig. 1c; [12]), the low concentrations of S-compounds in intermediate oxidation states [57, 58], and the chemosynthetic activity of *Campylobacterota* in the absence of O_2_ and NO_3_^−^ [27].

Nonetheless, our results need to be interpreted cautiously, because the underlying model is a simplification based on several assumptions. First, S^2−^ oxidation was assumed to occur at the upper boundary of euxinic water and above, based on the measured linear concentration profile of S^2−^, which indicated a diffusive flux towards the redoxcline without net production or consumption below [44]. The gross oxidation of S^2−^ within and below the redoxcline may in fact be higher, with a rate equal to that of S^2−^ production via SO_4_^2−^ reduction [13]. This would have led to a cryptic cycle not represented by the model. However, the finding that *sqr* expression fell off sharply below the redoxcline (110 m water depth), indicating negligible biological S^2−^ oxidation activity via the Sqr pathway, supports the assumption that S^2−^ oxidation proceeds at the upper boundary of euxinic water and above (Fig. 1e). Second, for the chemical oxidation, a realistic MnO_2_ concentration of 10 nM was assumed in the abiotic model. However, even a ten-fold higher concentration was still too low to reproduce the observed profile. The latter therefore implied rapid S^2−^ oxidation and thus a biologically catalyzed process. Lastly, other potential oxidants than MnO_2_ have been excluded in the laboratory experiments but might be present in undetectable concentrations in the Black Sea. However, this is rather unlikely based on the data presented in Fig. 1.

Estimation of the contribution of Mn-dependent S^2−^ oxidation to the total oxidation of S^2−^ requires a quantification of the downward-directed flux of MnO_2_ particles and the upward-directed flux of S^2−^. The downward-directed flux of particulate MnO_2_ should be equal to the upward-directed flux of Mn_diss._, because once the latter is oxidized to particulate Mn(IV), its movement is unidirectional, in the form of gravitational sinking [15, 20, 21]. For a Mn_diss._ concentration gradient of 0.32 mmol m^−3^ m^−1^, the corresponding flux would be in the range of 3.1 × 10^−7^–1.2 × 10^−6^ mmol Mn_diss._ m^−2^ s^−1^ (D_x_ of 1 or 4 × 10^−6^ m^−2^ s^−1^ [17]), although due to riverine inputs of Mn the real flux might be slightly higher [59]. The S^2−^ flux of 6.7 × 10^−7^–2.68 × 10^−6^ mmol S^2−^ m^−2^ s^−1^, based on a S^2−^ gradient of 0.67 mmol m^−3^ m^−1^ [9], is in agreement with the values reported by Brewer and Spencer (2.31 × 10^−6^ mmol S^2−^ m^−2^ s^−1^; [60]), Jørgensen et al. (5.1 × 10^−7^–3.3 × 10^−6^ mmol S^2−^ m^−2^ s^−1^; [58], calculated based on a diapycnal diffusivity coefficient of 1 or 4 × 10^−6^ m^−2^ s^−1^), and by Fuchsman et al. (1.24 × 10^−6^ mmol S^2−^ m^−2^ s^−1^; [61]). Assuming both the complete reduction of MnO_2_ to Mn(II) and the oxidation of S^2−^ to SO_4_^2−^ (and no quantitative importance of MnO_2_ reduction by Fe^2+^ [21]), roughly 25% of the total S^2−^ oxidation can be explained by MnO_2_. However, some of the electrons derived from S^2−^ oxidation are needed for chemosynthesis by *Sulfurimonas*. Based on the Mn(IV)/Mn(II) to S_2_O_3_^2−^/SO_4_^2−^ ratio of 3.7 determined in growth experiments with *Ca*. S. marisnigri SoZ1 [25], 7.5% of the electrons from S are used for CO_2_ reduction to support autotrophic growth, although larger proportions, up to 20%, have been reported [62]. Chemoautotrophic growth by Mn-reducing chemolithoautotrophic bacteria could therefore account for another 1–11% of the oxidation of the S^2−^ flux, depending on the assumed Mn flux and whether 7.5% or 20% of the electrons derived from S^2−^ are used for CO_2_ reduction. In summary, as much as one-third of the total S^2−^ flux may be utilized by chemolithoautotrophic Mn-dependent S^2−^ oxidation by *Sulfurimonas*.

### Lab-experiment-derived indications of S^2−^ oxidation with MnO_2_ by *Sulfurimonas* spp. in the Black Sea redoxcline

*Sulfurimonas* spp.-mediated S^2−^ oxidation with MnO_2_ in the Black Sea is further supported by the experimental data of Henkel et al. [25], obtained in an experiment in which the continuous addition of S^2−^ to MnO_2_-spiked medium inoculated with *Ca*. S. marisnigri SoZ1 resulted in bacterial growth and the accumulation of SO_4_^2−^. With Na_2_S addition reproducing a flux of 1 × 10^−3^ mmol S^2−^ m^−2^ s^−1^ or 2.5 × 10^−3^ mmol Na_2_S m^−2^ s^−1^, *Ca*. S. marisnigri SoZ1 abundance plateaued at 1.1 × 10^13^ or 2.2 × 10^13^ cells m^−3^, the equivalent of 3.4 × 10^−17^ or 11.6 × 10^−17^ mmol S^2−^ cell^−1^ m^−2^ s^−1^, respectively. In the Black Sea, the S^2−^ flux of 6.7 × 10^−7^–2.68 × 10^−6^ mmol S^2−^ m^−2^ s^−1^ from euxinic water feeds the S^2−^-oxidizing community at the upper boundary of euxinic water. At a cell density of 4 × 10^10^ *Sulfurimonas* spp. cells m^−3^ (Fig. 5a), a single *Sulfurimonas* spp. cell is fed by a S^2−^ flux of 1.68 to 6.70 × 10^−17^ mmol S^2−^ m^−2^ s^−1^. The overall order of magnitude of the S^2−^ flux needed to feed a single cell in lab experiments and in the Black Sea was surprisingly similar, indicating that the natural abundance of *Sulfurimonas* spp. is consistent with the observed input flux of S^2−^ and MnO_2_.

### Elevated abundance of *Sulfurimonas* spp. as an indicator of the quantitative importance of Mn-dependent S^2−^ oxidation in the Black Sea redoxcline

The present study identified *Sulfurimonas* spp. as a key player in S^2−^ oxidation in the redoxcline of the Black Sea, but their activity may not explain total S^2−^ consumption. Along with *Campylobacterota* or *Sulfurimonas* spp., gammaproteobacterial sulfur oxidizers (GSO) of clades SUP05 and BS-GSO2 may contribute quantitatively to S^2−^ oxidation in the Black Sea [13, 27, 61, 63, 64]. In the isolate *Candidatus* Thioglobus autotrophicus EF1 (clade SUP05), S oxidation is coupled to the incomplete reduction of NO_3_^−^ to NO_2_^−^ [65], in agreement with the lack of nitrite reductases in its genome. Those genes are also absent in the metagenome-assembled-genome (MAG) of *Ca*. Thioglobus pontius (SUP05 from the Black Sea redoxcline) [13]. The Black Sea MAG of *Ca*. Thioponita autotrophica of clade BS-GSO2 indicates the genetic potential to reduce NO_3_^−^ and NO_2_^−^ based on the presence of nitrate reductase (*narGHI*) and nitrite reductase (*nirBD/K*) genes [13]. However, whether SUP05 or BS-GSO2 also utilizes MnO_2_ as a terminal electron acceptor is unknown.

SUP05 are considered to be non-motile [66], evidenced by microscopic inspection and a lack of genes encoding flagella; however, this evidence is inconclusive (Robert Morris, personal communication) and no data are available for clade BS-GSO2. Our results lead to the hypothesis that *Sulfurimonas* spp. dominates the S-oxidizing community in the Black Sea when a stable Mn cycle has developed that separates O_2_, NO_3_^−^, and NO^2−^ from S^2−^. A larger role for *Sulfurimonas* spp. than for BS-GSO2 and SUP05 would then be based on the ability to perform active movement and reduce MnO_2_, which would favor the success of these bacteria in the presence of a well-developed Mn cycle. Such conditions might be reflected in the accumulation of Mn_react._ (Mn^3+^) at pelagic redoxclines, the formation of which requires a hydrographically stable water column without major disturbances such as by lateral intrusions [1]. Whether the remaining S^2−^ oxidation can be explained by the activity of phototrophs [67], the vertical migration of magnetotactic bacteria with internal vacuoles [9], or by as-yet-unidentified mechanisms is unknown.

### General implications for geochemical cycles and microbial communities in redoxclines

Microbially catalyzed Mn-dependent S^2−^ oxidation affects the depth of the S^2−^ interface (Fig. 5), thereby fostering the separation of S^2−^ from O_2_, NO_3_^−^ and NO_2_^−^. This separation has consequences for element cycling, e.g., the N-loss processes of denitrification and anammox. In the central Black Sea, where heterotrophic denitrification was generally undetectable within the redoxcline and denitrification via S^2−^ oxidation is excluded due to a missing interface between S^2−^ and NO_3_^−^, anammox was the main N-loss process [68].

Although the Black Sea redoxcline is the most prominent system with a zone that simultaneously lacks O_2_ and S^2−^, other examples from euxinic systems have been reported. In the central Baltic Sea, Hannig et al. [69] showed a shift in the N-loss process from S^2−^-coupled denitrification to anammox in the redoxcline after saltwater inflows in 2002 and 2003. In 2005, after re-establishment of the redoxcline, a Black Sea-like separation of O_2_, NO_3_^−^, and NO_2_^−^ from S^2−^ was detectable that was accompanied by a 3- to 13-fold increase in the maximum concentrations of reduced and oxidized Mn within the redoxcline. Under these conditions, anammox instead of denitrification was the main N-loss process. The authors suggested that Mn-dependent S^2−^ oxidation, with the subsequent vertical separation of NO_3_^−^ and S^2−^, supported anammox rather than denitrification as the dominant N-loss process. Similar indication came from marine sediments. Engström et al. [70] found that the relative contribution of anammox to total N_2_ production increased with Mn-oxide content to up to 80%, indicating that Mn-oxides compete with denitrification for substrates, thereby favoring anammox activity. It can therefore be suggested that Mn-cycling eliminates the inhibitory effect of S^2−^ on anammox [71].

Pelagic redoxclines have been reported from stratified systems worldwide, ranging from meromictic and seasonally anoxic lakes (e.g., Fayetteville Green Lake and Lake Dagow [1, 72]) to brackish (e.g., Baltic and Black Seas [10]), marine (e.g., Cariaco basin and anoxic fjords [2, 3]), and hypersaline (Orca basin [4]) environments. Despite fundamental differences in the general conditions and patterns of their chemical redox profiles [73], those systems have substantial similarities with respect to the biogeochemical processes at their pelagic redoxclines. A prominent example is the cycling of dissolved and particulate Mn species in the presence of sulfide, which suggests that those redoxclines host microbial communities with a similar niche as reported here for the Black Sea genus *Sulfurimonas*. Thus, chemosynthesis in the absence of NO_3_^−^ and O_2_ was already reported, with microbial S^2−^ oxidation coupled to Mn reduction proposed in the Cariaco Basin and in the Baltic Sea [2, 74]. It is therefore not surprising that another Mn-reducing and S^2−^ oxidizing species, *Ca*. Sulfurimonas baltica GD2, could be isolated from the bottom waters of the Gotland Basin in the Baltic Sea [26].

## Conclusions

In this study, the geochemistry of the pelagic redoxcline of the Black Sea was characterized at high resolution. The local abundance and gene expression of *Sulfurimonas* spp. were determined as well, together with laboratory-based assessments on Mn-dependent S^2−^ oxidation rates by *Ca*. S. marisnigri SoZ1. *Sulfurimonas* spp. were highly abundant across the redoxcline, where O_2_, NO_3_^−^, and NO_2_^−^ were absent. The expression of *sqr*, *soxCDYZH*, *soxXYZAB*, *psrABC*, and *sorAB* at the upper boundary of euxinic water indicated the complete oxidation of S^2−^ and S-intermediates to SO_4_^2−^ by *Sulfurimonas* spp., without an important role for S_2_O_3_^2−^. The cellular abundance of *Sulfurimonas* spp. was consistent with the availability of MnO_2_ and with S^2−^ fluxes and suggested the coupling of S^2−^ oxidation to MnO_2_ by this genus. The rapid oxidation of S^2−^ at the upper boundary of euxinic water was crucial in explaining the observed S^2−^ concentration profile. Abiotic oxidation of S^2−^ with MnO_2_ was too slow to counterbalance S^2−^ fluxes from euxinic water. By contrast, the rate of biologically catalyzed oxidation was sufficiently high and consistent with the expression of the *sqr*, which *Sulfurimonas* spp. dominated by about 90 percent at the boundary to euxinic water. Biological S^2−^ oxidation with MnO_2_ can explain the accumulation of Mn^3+^ and offers a plausible explanation for the chemosynthetic activities of *Campylobacterota* in the Black Sea in the absence of O_2_, NO_3_^−^, and NO_2_^−^ [27]. Our study therefore suggests that, by catalyzing the oxidation of S^2−^ with MnO_2_, *Sulfurimonas* spp. in the Black Sea redoxcline play a crucial role in the establishment and maintenance of a broad anoxic and non-sulfidic zone separating oxic and euxinic water. This may in turn create ecological niches for other important functional groups of prokaryotes, such as anammox bacteria, that thrive in these zones.

Because the modern Black Sea is a prime example of a redox-stratified aquatic ecosystem, we are confident that our findings are of relevance for other stratified settings worldwide, including lakes, fjords, and semi-restricted brackish/marine basins. As the first indication for biological Mn-dependent S^2−^ oxidation came from marine sediments [75, 76], the results of the present work may also be relevant for marine sediments, where rapid biological Mn-dependent S^2−^ oxidation could prevent the escape of sulfide into bottom waters, thereby counteracting the spread of hypoxia.

## Supplementary information

Supplementary material and methods

Supplementary tables S1–S4

Supplementary file manual list of sulfur oxidation genes

Supplementary file standards for RNA quantification

Supplementary file numerical model

## Data Availability

Metadata for the research cruise can be found at IOWMeta with the identifier MSM33 and under 10.2312/cr_msm33. The contigs and CDS used for the present work are available via DOI 10.12754/data-2021-0005. Sequence data for this study have been deposited in the European Nucleotide Archive (ENA) at EMBL-EBI using the data brokerage service of the German Federation for Biological Data (GFBio [39]), in compliance with the Minimal Information about any (X) Sequence (MIxS) standard [40]. Raw sequence data for the metagenome, metatranscriptome, and amplicon data were deposited under umbrella project PRJEB46990 with the accession numbers PRJEB46962, PRJEB46963, and PRJEB46963, respectively. The spreadsheet of the Excel-based numerical model and the manual list of S oxidation genes are available in the supplement.
