# Supplementary material for: Biological manganese-dependent sulfide oxidation impacts elemental gradients in redox-stratified systems: indications from the Black Sea water column
Source: ISME J. 2022 Feb 5;16(6):1523–33. doi: 10.1038/s41396-022-01200-3 (PMC9122950; doi:10.1038/s41396-022-01200-3)
Supplement: Supplementary file 1 — Supplementary material and methods [file 41396_2022_1200_MOESM1_ESM.docx]

**Supplementary material and methods to:**

Biological manganese-dependent sulfide oxidation impacts elemental gradients in redox stratified systems: Indications from the Black Sea water column

**Material and methods**

**Field sampling**

Samples were taken during a cruise of the R/V “Maria S. Merian” (MSM33) in November/December 2013, with sampling performed as described previously [1]. All data in the current study originated from station 32, located in the western central gyre of the Black Sea (43° 31.922′ N, 32°30.909′ E; water depth 2070 m). Additionally, high-resolution data of total dissolved Mn (Mn_diss_) and dissolved reactive Mn (dMn_react_; [2]) were obtained from station 66, in the eastern central gyre (43° 31.8019′ N, 36° 05.9960′ E; total water depth 2177 m). Temperature, pH, and salinity were profiled using a continuous pump-CTD-system [3]. Water samples for microbial community analyses and determinations of cell numbers were collected in free-flow bottles (Hydrobios) and, for metatranscriptomic samples, using an automatic flow injection sampler (AFIS [4]) attached to a CTD-rosette. *In situ* fixation in the AFIS system provides relatively unbiased gene expression data from oxygen-deficient water layers [4]. To construct water column profiles from successive casts, the data were aligned according to the density of cast P0014F13 (station 32), as described in Schulz-Vogt et al. [1].

**Gases, nutrients, and metals**

Data for the water column profiles of dissolved gases (O_2_, H_2_S) and nutrients (NO_3_^−^, NO_2_^−^, NH_4_^+^) were extracted from the profiles reported in Schulz-Vogt et al. [1]. Contamination by ambient air was avoided, as the gas sensors and nutrient autoanalyzer were directly connected to the tubing of the pump-CTD-system. Water samples for Mn_diss._ and dMn_react._ [2] were taken directly from the pump-CTD water flow using 10-ml PE syringes. Two ml of the water was immediately filtered through 0.45-μm syringe filters (SFCA), stored in 2-ml reaction tubes, and acidified to 2 vol.% HNO_3_. The remaining water sample was stored in the syringe in the dark for 36 h and then similarly filtered and acidified. The concentrations of both dissolved Mn fractions were measured by inductively coupled plasma-optical emission spectroscopy (ICP-OES, iCAP 6400 duo, Thermo Fisher Scientific) using matrix-matched external calibration and Sc as the internal standard. Precision and trueness were checked against the international reference material SLEW-3 (NRCC; spiked with 1.82 µM Mn) and were 1.1% and −0.1%, respectively [5]. The concentration of dMn_react._, which is assumed to mainly consist of dissolved Mn^3+^, was calculated from the difference in Mn_diss._ before and after 36 h of storage [2, 6]. For particulate Mn (Mn_part_), 2 l of seawater obtained from CTD bottles of the same cast was filtered through 0.4-μm polycarbonate filters. The filters were then rinsed with 18.2MΩ cm water to remove salt, stored frozen in Petri dishes, and finally dried at 40°C for 2 days. The filters were placed in Teflon vessels and 1.5 ml of HClO_4_ was added to promote their decomposition, followed by the addition of 1 ml of HF to digest the suspended particulate matter in closed vessels at 180°C for 12 h. Following digestion, the acids were evaporated to near dryness and the samples were fumed three times with 2 ml of 6 M HCl before they were finally diluted to 4 ml with 2 vol% HNO_3_. Particulate Mn was measured by ICP-OES using external calibration; Sc served as the internal standard. Precision and trueness, checked with the international reference material SGR-1b, were 0.6% and +2.0%, respectively [5].

**Catalyzed reporter deposition fluorescence *in situ* hybridization (CARD-FISH)**

CARD-FISH was carried out according to the protocols of Pernthaler et al. [7] and Sekar et al. [8] , modified as described in [9]. Samples were fixed with formaldehyde (final concentration 2%) for 6-12 h. 50 ml fixed sample were filtered on 0.2 µm polycarbonate filters and stored at -80°C until analysis. For the enumeration of *Bacteria*, a mixture of horseradish-peroxidase-labeled oligonucleotide probes EUB338 [GCTGCCTCCCGTAGGAGT], EUB338-II [GCAGCCACCCGTAGGTGT], and EUB338-III [GCTGCCACCCGTAGGTGT] [10] was used. *Epsilonproteobacteria* were enumerated using probe EPSY914 [GGTCCCCGTCTATTCCTT] [11]. Non-specific binding was determined using the NonEUB probe [ACTCCTACGGGAGGCAGC] [12]. Signal amplification was achieved using tyramides labeled with Alexa488 (Invitrogen). Filter sections were counterstained with DAPI (1 mg ml^−1^) and inspected using an epifluorescence microscope (Axioscope, Carl Zeiss) together with filter sets 01 (DAPI) and 10 (Alexa 488). For manual evaluation of the fractions of hybridized cells, between 500 and 1000 DAPI-stained cells in randomly distributed microscopic fields were counted for each filter section. In both DAPI-stained and hybridized samples, bacteria are usually counted with a standard deviation of < 7.5% for replicate samples [9].

**Sampling, processing, and analysis of the metagenomic and metatranscriptomic data**

**Amplicon sequencing of the 16S rRNA gene and 16S rRNA**

For rRNA analysis, 1 l of water was filtered on 0.2 µm Durapore polyvinylidene fluoride filter (Millipore), flash frozen in liquid nitrogen and stored at -80 °C until nucleic acid extraction. Samples for 16S rRNA gene and 16S rRNA amplicon sequencing were extracted using the AllPrep DNA/RNA kit (Qiagen; Hilden, Germany). DNA extracts were stored directly whereas from the extracted RNA a maximum of 100 ng was DNase treated using the Turbo DNA-free kit (Thermo Fisher Scientific; Waltham, MA, USA). From the DNase-treated RNA, a maximum of 20 ng was subsequently reverse transcribed with Multiscribe RT (Thermo Fisher Scientific). All samples of DNA and treated RNA, as well as additional Mock Community samples (Zymo Research; Freiburg, Germany) as controls, were sent to LGC Genomics (Berlin, Germany) for sequencing with MiSeq (Illumina). Here, the hypervariable V3-V4 region of the 16S rRNA sequence was amplified in DNA extracts and treated RNA (cDNA) of all samples with the universal primers U341F (5՚-CCTAYGGGRBGCASCAG-3՚) and U806R (5՚-GGACTACNNGGGTATCTAAT-3՚) [13]. The PCRs included about 1-10 ng of DNA extract and cDNA (total volume 1 µL), respectively, 15 pmol of each forward primer and reverse primer in 20 µL volume of 1 x MyTaq buffer containing 1.5 units MyTaq DNA polymerase (Bioline GmbH) and 2 µl of BioStabII PCR Enhancer (Sigma-Aldrich). For each sample, the forward and reverse primers had the same 10-nt barcode sequence. PCRs were carried out for 30 cycles using the following parameters: 1 min 96°C pre-denaturation, 96°C denaturation for 15 s, 55°C annealing for 30 s, 70°C extension for 90 s, hold at 8°C. Concentrations of the 16S amplicon were determined by gel electrophoresis. About 20 ng amplicon DNA of each sample were pooled for up to 48 samples carrying different barcodes. The amplicon pools were purified with one volume Agencourt AMPure XP beads (Beckman Coulter) to remove primer dimer and other small mispriming products, followed by an additional purification on MiniElute columns (Qiagen). About 100 ng of each purified amplicon pool DNA was used to construct Illumina libraries using the Ovation Rapid DR Multiplex System 1-96 (NuGEN). Illumina libraries were pooled and size selected by preparative gel electrophoresis. Sequencing was done on an Illumina MiSeq using V3 Chemistry (Illumina) with paired-end reads of 2 x 300 bp. The resulting sequences were analyzed using the SILVA_NGS pipeline (release 138.1) [14, 15], with settings and OTU clustering based on 97% similarity, as described previously [16].

**Metagenomic and metatranscriptomic analyses**

Samples for the metagenome analyses were obtained from four different water depths using conventional CTD-rosette sampling. Samples for metatranscriptome analyses were collected from six depths spanning the redoxcline, from the oxic to the sulfidic zone, using an automatic flow injection sampler (AFIS) allowing *in situ* fixation of the 2.7 l water samples [17]. RNA from the filtered samples was extracted as previously reported [18]. Two internal RNA standards were added at the beginning of the RNA extraction for the absolute quantification of transcripts l^-1^ in the downstream analyses [19]. These standards were obtained after linearizing the plasmid pFN18A HaloTag T7 Flexi (Promega, Wisconsin, USA) as well as the plasmid pTXB1 (ThermoFisher, Massachusetts, USA) and transcribing a 970 bp / 914 bp part of it into RNA using the MEGAscript T7 Transcription Kit (ThermoFisher, Massachusetts, USA). The sequence of the used standards is given in the supplementary material.

Due to a lysis buffer spill during extraction of the sample obtained at 111 m water depth, absolute transcript numbers in that sample are slightly overestimated. DNA for metagenome analysis was extracted using the QIAmp DNA mini kit (Qiagen). The purified RNA was sent to Fasteris (Plan-les-Ouates, Switzerland) for Illumina-HiSeq sequencing after rRNA depletion using the RiboZero kit for bacteria (Epicentre, Madison, WI, USA).

After removal of rRNA reads as well as quality and adaptor clipping, read pairs from four metagenome libraries were combined and assembled using idba_ud 1.1.1 [20].

Non protein coding rRNA or the internal standards reads were removed using the SortMeRna software (v1.9, [21]) against default reference rRNA sequences [21] as well as sequence data from the internal standards (see supplementary material). These noncoding RNA sequences were subsequently screened for internal standards via a LAST search (minimum score: e = 500) [22] in order to retrieve the number of reads encoding the added standards.

Gene calling was performed on contigs > 200 nt using Prodigal's meta-procedure [23]. Predicted CDSs were functionally annotated using KEGG (blastp with blast-score-ratio [BSR] [24] cut-off of 0.4), Interproscan V5.25 (with default settings) [25], and eggnog-mapper (default settings on the bacterial and archaeal datasets) [26]. Predicted CDS were further compared by similarity using blastp against NCBI’s NR database and a manually generated database from Uniprot and Swissprot entries (available as supplementary material). Single-end metatranscriptome reads were also adaptor-clipped using mira 4.0.2 [27] and quality clipped using sickle 1.33 (default settings) [28], mapped onto the metagenome assembly using bowtie2 2.2.4 [29] with the very-sensitive settings, and summarized using featureCounts 1.4.6 [30], requiring a minimum of 20 overlapped bases to assign a read to a feature.

The number of metatranscriptome reads coding for internal standards was determined and used to estimate the absolute number of transcripts l^−1^, as detailed in Satinsky et al. [19].

The identified genes (see below) were taxonomically annotated using CAT (v5.2.3), based on the Diamond aligner (v2.0.8.147 [31]) in blastp mode against NCBI’s NR database (as of July 1, 2021) with the r-parameter set to 3 [32]. To ensure maximum sensitivity, all CDS which contained annotated genes relevant to this study (*sqr*, *soxCDYZH*, *soxXYZAB*, *psrAB* or *sorAB*) were additionally run with NCBI blast in blastp mode and used again for CAT taxonomy. No differences in taxonomic affiliation of the shown genes were observed. As indicated by the 16S rRNA gene taxonomy (described above), all *Campylobacterota* reads belonged to the order *Campylobacterales*, with families *Arcobacteraceae*, Rs-M59 termite group, *Sulfurospirillaceae*, and *Helicobacteraceae* (synonyms *Thiovulaceae*, *Sulfurovaceae*, and *Sulfurimonadaceae* – <http://lpsn.dsmz.de>). Reads of the Rs-M59 termite group and of *Sulfospirillaceae* were detectable only in rare cases, restricted to samples close to oxic waters (referred to as “others” in Fig. 2 and Tab. S1-S4), and always < 0.5%. Within the family *Helicobacteraceae*, *Sulfurimonas*-related reads contributed 99.9–100%. Thus, all gene transcripts with a taxonomic annotation to the family *Helicobacteraceae* were assigned to *Sulfurimonas*.

Genes annotated as *Sulfurimonas* based on the criteria detailed above were extracted, and sulfur oxidation genes were identified based on functional annotation, sequence similarity, and gene synteny. The following gene transcripts were identified by annotation with KEGG orthology (KEGG and gene identifiers): *sqr* (K17218; BSMG_1655_11, BSMG_2166_10, BSMG_711_9, BSMG_771_1), *soxX* (K17223; BSMG_40400_1, BSMG_10760_3), *soxY* (K17226; BSMG_40400_2, BSMG_18319_1, BSMG_10760_4, BSMG_1655_16), *soxZ* (K17227; BSMG_40400_3, BSMG_35836_3, BSMG_1655_17, BSMG_10760_5), *soxA* (K17222; BSMG_124729_1, BSMG_35836_2), *soxB* (K17224; BSMG_37923_1, BSMG_35836_1, BSMG_241760_1, BSMG_241863_1, BSMG_30614_1), *soxC* (K17225; BSMG_1655_14) and *sorA* (K07147; BSMG_2930_6), with the identifier BSMG (Black Sea metagenome) followed by the contig number and then by the gene number on the respective contig (available via doi). C-type cytochrome *soxD* (BSMG_1655_15) was identified based on gene synteny with *soxC* (BSMG_1655_14) and *soxY* (BSMG_1655_16) according to the *soxCDYZH* organized cluster found in *Sulfurimonas* spp. [33–35] and the annotation as a c-type cytochrome by KEGG (K08738) and InterProScan (IPR003088, IPR009056). BSMG_4011_1 and BSMG_3786_1 were identified as *soxZ* using InterProScan (IPR014880). BSMG_3786_2 was identified as *soxH* based on gene synteny with soxZ (BSMG_3786_1), a BSR of 0.71 against *soxH* from *Sulfurimonas gotlandica* GD1^T^ (Uniprot entry B6BGQ4_SULGG), and beta-lactamase like annotation by InterProScan (IPR001279). BSMG_2930_7 was identified as *sorB* based on gene synteny with *sorA* (BSMG_2930_6), annotation as cytochrome c oxidoreductase subunit b by eggnog (05WJY), and a BSR of 0.43 against sulfite:cytochrome c oxidoreductase subunit B from *S. gotlandica* GD1^T^ (H1FYW1_SULGG). Polysulfide reductase genes (*psrACB*) were identified based on gene synteny (BSMG_414_19, BSMG_414_20, BSMG_414_21), BSR (0.87, 0.79, 0.87) against *psrACB* from *S. gotlandica* GD1^T^ (B6BMI1_SULGG, B6BMI0_SULGG, B6BMH9_SULGG), and identical protein domains found by InterProScan when compared with the following curated Swissprot entries of *psrACB* from *Wolinella succinogenes* (P31075; P31076; P31077): *psrA* (IPR006311, IPR006656, IPR006657, IPR006963), *psrC* (IPR005614) and *psrB* (IPR017896, IPR017900). *S. gotlandica* GD1^T^ was chosen as the reference because it is the closest cultured relative species of *Ca.* S. marisnigri SoZ1 [36]. In the event that annotation based on CAT taxonomy was restricted to the order level (*Campylobacterales*), the whole contig was inspected manually and taxonomic annotation of other genes in the same contig provided a better taxonomic resolution (family level or better).

**Estimation of the rates of S^2-^ oxidation with MnO_2_ by *Ca.* S. marisnigri SoZ1**

The rates of biological S^2-^ oxidation with MnO_2_ by the isolate *Ca.* S. marisnigri SoZ1 (= JCM 39139; = DSM 111879) were determined as follows: six glass bottles were filled with 500 ml of anoxic medium [37], supplemented with 20 mM MnO_2_ (Merck, Darmstadt, Germany, finely ground with an agate ball mill), and closed with butyl rubber stoppers. Three bottles were inoculated with *Ca.* S. marisnigri SoZ1 [36] and 2 mM thiosulfate (Na_2_S_2_O_3_), while the others remained sterile (controls), without the addition of Na_2_S_2_O_3_ because preliminary tests showed the chemical reaction of S^2−^ with S_2_O_3_^2−^, in agreement with the work of Siu & Jia [38]. All bottles were constantly shaken at 10°C in the dark for 12 days. After 12 days of incubation, S_2_O_3_^2−^, determined following the method described by Henkel et al. [37], was undetectable in all culture replicates, which ensured that no S_2_O_3_^2−^ affected the rate estimations. H_2_S and pH microsensors were placed in the constantly stirred medium at 10°C. To avoid O_2_ contamination, the headspace was constantly flushed with argon gas. The consumption of eight (culture) or five (control) single additions of a 10 mM Na_2_S stock solution (~30 µM S^2-^ in medium) was recorded. One replicate of the culture and one of the control were treated with 20 mM sodium azide (data not shown), whereas the other two replicates were pasteurized at 80°C for 1 h and then cooled to 10°C. Poisoned replicates were incubated at 10°C for 1 h. After treatment of the samples with azide or heat, the consumption of another three Na_2_S additions was recorded. The cellular abundance of *Ca.* S. marisnigri SoZ1 before the start of the experiment was determined by epifluorescence counting with DAPI as described in [37]. Cell abundances were 5.87 × 10^7^ (replicate 1), 4.71 × 10^7^ (replicate 2), and 5.10 × 10^7^ (replicate 3) cells ml^−1^.

According to chemical reaction kinetics, the second-order chemical reaction of MnO_2_ and S^2-^ can be forced into a chemical reaction of pseudo-first order kinetics by adding MnO_2_ in excess [39]. In that case, the speed of the reaction depends only on the concentration of one reactant, here S^2−^, which can be easily measured at high temporal resolution using a H_2_S microsensor. The addition of 20 mM MnO_2_ allowed the abiotic reaction to occur at maximum speed and to be reproducibly constant with respect to temperature and pH. The rate can easily be subtracted from the overall reaction rate, described by Eq. 2.

(2) $\left[ S^{2-} \right]_{t}=\left[ S^{2-} \right]_{0} e^{(a+kt)}$

Using normalized data of the S^2−^ concentration, Eq. 2 can be transposed to Eq. 3.

(3) $\frac{\left[ S^{2-} \right]_{t}}{\left[ S^{2-} \right]_{0}}=e^{(a+kt)}$

where [S^2−^]_t_ is the concentration of S^2-^ at time t; [S^2−^]_0_ is the initial concentration of S^2−^, as a correction variable for the y-intercept; *k* is the reaction rate coefficient (s^−1^); and t is the time in seconds. A non-linear least-squares fit to Eq. 3 was performed with R (version 3.5.1) for individual spikes of Na_2_S to determine the overall reaction rate coefficient *k*. In the absence of biological S^2-^ oxidation, the value of *k* should be identical to that of a purely chemical reaction, reflected by the control measurements.

The biological reaction rate (*k*_bio_) was calculated as the difference between *k* after pasteurization and the overall reaction rate coefficient *k* before pasteurization. After pasteurization, oxidation rates in the sterile controls and in the biological treatments were indistinguishable, which showed that heat inhibited the biological S^2-^ oxidation completely and that the amount of MnO_2_ was sufficiently high to ensure pseudo-first-order reaction rate kinetics, despite the presence of less MnO_2_ in the treatment with *Ca.* S. marisnigri SoZ1 after 12 days of growth. *k*_bio_ was divided by the cellular abundance of *Ca.* S. marisnigri SoZ1 to obtain a cell-specific reaction rate coefficient (*k*_cell_), with the mean value (*k*_cell_ = −1.05 × 10^−13^ cell^−1^ s^−1^) used for numerical modeling. The cellular reaction rate coefficient *k*_cell_ was multiplied by the abundance of *Sulfurimonas* spp. (cells l^−1^) and the ambient S^2-^ concentration (µM) to calculate the local S^2−^ oxidation rate (µM s^−1^).

**Sensor calibration.** The Unisense sensor system consisted of a pH sensor with internal reference (50-µm tip size), an H_2_S sensor (50-µm tip size), and a microsensor multimeter. The pH sensor was calibrated with three pH buffer solutions (pH 4.01, pH 7, pH 10), and the H_2_S sensor with a series of S^2-^ concentrations (0–30 µM in 5-µM steps) in pH 3 buffer solution (1.5 M potassium hydrogen phthalate in 0.25% HCl). Subsamples were analyzed spectrophotometrically according to the Cline method [40] for additional control of the calibration series. Data were collected using the software SensorTrace Logger from Unisense. H_2_S concentrations measured in the experiment were corrected for temperature, salinity, and pH, according to the manufacturer’s manual, to obtain S^2−^ values.

**Growth of *Ca.* S. marisnigri SoZ1 with S^2−^ and MnO_2_ in a semi-continuous culturing approach**

The semi-continuous culture experiment was performed by Henkel et al. [37] to identify the reaction end products of MnO_2_ and S^2−^ both under sterile conditions and in reactions containing *Ca.* S. marisnigri SoZ1. Due to length restrictions of the journal, only a brief description was possible. In the experiment, the cellular abundance of *Ca.* S. marisnigri SoZ1 was proportional to the applied S^2−^ flux and the results added important information to the data presented in this study. Three 1-l glass bottles were filled with 500 ml of anoxic medium and connected with gas-tight tubing (Fluran HCA, ISMATEC) pierced through butyl rubber stoppers with glass pipes. One bottle was spiked with 10 mM Na_2_S (final concentration), one with 10 mM MnO_2_ (abiotic control), and one with 10 mM MnO_2_ and later *Ca.* S. marisnigri SoZ1. Sterile medium from the Na_2_S spiked bottle was pumped simultaneously (3 µl min^−1^) into the culture and control bottles via a multi-channel peristaltic pump. The length and diameter of the pump tubing were identical to ensure the equal addition of S^2−^ to the control and biological treatments. The experiment was run for 2 days before pre-grown cells (~1 × 10^5^ cells ml^-1^) were added to the culture treatment. After the cellular abundance had reached a plateau (day 12), the flow of 10 mM Na_2_S was increased from 3 µl min^−1^ to 7.5 µl min^−1^.

**Modeling the S^2-^ concentration profile of the Black Sea**

The abundance and activity of *Sulfurimonas* spp. in the Black Sea was combined with the S^2−^ oxidation rates of the representative isolate *Ca.* S. marisnigri SoZ1 as determined in lab experiments. The quantitative impact of S^2−^ oxidation by *Sulfurimonas* spp. on the geochemical water column profile in the Black Sea was estimated by combining the results from the field study with those of the lab experiments using a numerical model. In the modeling approach of Schulz, 2006 [41], the differential equation for diffusive transport (Fick's second law) is described by Eq. 4:

(4) δc / δt = D δ^2^c / δx^2^

where D is the diffusion coefficient; c is the concentration; t is the time; and x is the distance coordinate. The equation is solved using the explicit numerical solution obtained with Eq. 5, adapted from Schulz-Vogt et al. [1]:

(5) $C_{S^{2-}(x,t+\Delta t)}=C_{S^{2-}(x,t)}+\frac{\Delta t \times D_{x} \times(C_{S^{2-}\left( x+\Delta x,t \right)}-2 \times C_{S^{2-}\left( x,t \right)}+C_{S^{2-}\left( x-\Delta x,t \right)})}{{\Delta x}^{2}}+$

(5.1, biological) $\Delta t \times k_{cell} \times{CA}_{EPSY914\left( x \right)}\times C_{S^{2-}\left( x,t \right)}\times F_{Sulfurimonas in Campylobacterota}$

(5.2, chemical) $\Delta t \times k_{chem} \times C_{S^{2-}(x,t)}$

where C_S_^2−^ is the concentration of S^2−^ at a given water depth x and time t; D_x_ is the diapycnal diffusivity at water depth x, as determined by Gregg and Yakushev (we choose 4 × 10^−6^ m^2^ s^−1^ or 1 × 10^−6^ m^2^ s^−1^ as upper and lower limit of diapycnal diffusivity); [42]). The consumption of S^2−^ via biological or chemical oxidation was taken into account in the diffusional term (Eq. 5) by Eq. 5.1 or Eq. 5.2, respectively. Biological S^2−^ oxidation was calculated by multiplying the cell-specific reaction rate coefficient of *Ca.* S. marisnigri SoZ1 (*k*_cell_ = −1.05 × 10^-13^ l cell^−1^ s^−1^) by the cellular abundance of *Sulfurimonas* spp. and the local S^2−^ concentration. Chemical S^2−^ oxidation was calculated using a chemical reaction rate coefficient (*k*_chem_ = −9.53 × 10^−8^ s^−1^, after Yao and Millero [39] based on a constant MnO_2_ concentration of 10 nM, pH 7, and 10°C) and the local S^2−^ concentration. The starting condition of the model were 10 µM S^2−^ at 120 m water depth. Oxidation was assumed at the chemocline (106 m water depth) and above based on the vertical concentration profile of S^2−^ (see the Discussion for details). The Excel-based spreadsheet of the model is provided as supplementary material.

**Data availability**

Metadata for the research cruise can be found at IOWMeta with the identifier MSM33. Contigs and CDS used for the present work are available via DOI 10.12754/data-2021-0005. Sequence data for this study have been deposited in the European Nucleotide Archive (ENA) at EMBL-EBI using the data brokerage service of the German Federation for Biological Data (GFBio [43]), in compliance with the Minimal Information about any (X) Sequence (MIxS) standard [44]. Raw sequence data for the metagenome, metatranscriptome, and amplicon data were deposited under umbrella project PRJEB46990 with the accession numbers PRJEB46962, PRJEB46963, and PRJEB46963, respectively. The spreadsheet of the Excel-based numerical model and the manual list of S oxidation genes are available as supplementary material.

1. Schulz-Vogt HN, Pollehne F, Jürgens K, Arz HW, Bahlo R, Dellwig O, et al. Effect of large magnetotactic bacteria with polyphosphate inclusions on the phosphate profile of the suboxic zone in the Black Sea. *ISME J* 2019; **13**: 1198–1208.

2. Schnetger B, Dellwig O. Dissolved reactive manganese at pelagic redoxclines (part I): A method for determination based on field experiments. *J Mar Syst* 2012; **90**: 23–30.

3. Strady E, Pohl C, Yakushev E V., Krüger S, Hennings U. PUMP–CTD-System for trace metal sampling with a high vertical resolution. A test in the Gotland Basin, Baltic Sea. *Chemosphere* 2008; **70**: 1309–1319.

4. Feike J, Jürgens K, Hollibaugh JT, Krüger S, Jost G, Labrenz M. Measuring unbiased metatranscriptomics in suboxic waters of the central Baltic Sea using a new in situ fixation system. *ISME J* 2012; **6**: 461–470.

5. Dellwig O, Wegwerth A, Schnetger B, Schulz H, Arz HW. Dissimilar behaviors of the geochemical twins W and Mo in hypoxic-euxinic marine basins. *Earth-Science Rev* 2019; **193**: 1–23.

6. Dellwig O, Schnetger B, Brumsack H-J, Grossart H-P, Umlauf L. Dissolved reactive manganese at pelagic redoxclines (part II): Hydrodynamic conditions for accumulation. *J Mar Syst* 2012; **90**: 31–41.

7. Pernthaler A, Pernthaler J, Amann R. Fluorescence in situ hybridization and catalyzed reporter deposition for the identification of marine bacteria. *Appl Environ Microbiol* 2002; **68**: 3094–3101.

8. Sekar R, Pernthaler A, Pernthaler J, Warnecke F, Posch T, Amann R. An improved protocol for quantification of freshwater Actinobacteria by fluorescence in situ hybridization. *Appl Environ Microbiol* 2003; **69**: 2928–2935.

9. Grote J, Labrenz M, Pfeiffer B, Jost G, Jürgens K. Quantitative distributions of *Epsilonproteobacteria* and a *Sulfurimonas* subgroup in pelagic redoxclines of the central Baltic Sea. *Appl Environ Microbiol* 2007; **73**: 7155–7161.

10. Daims H, Bruhl A, Amann R, Schleifer K, Wagner M. The domain-specific probe EUB338 is insufficient for the detection of all Bacteria: Development and evaluation of a more comprehensive probe set. *Syst Appl Microbiol* 1999; **22**: 434–444.

11. Grote J, Jost G, Labrenz M, Herndl GJ, Jürgens K. *Epsilonproteobacteria* represent the major portion of chemoautotrophic bacteria in sulfidic waters of pelagic redoxclines of the Baltic and Black Seas. *Appl Environ Microbiol* 2008; **74**: 7546–7551.

12. Wallner G, Amann R, Beisker W. Optimizing fluorescent in situ hybridization with rRNA-targeted oligonucleotide probes for flow cytometric identification of microorganisms. *Cytometry* 1993; **11**: 136–143.

13. Sundberg C, Al-Soud WA, Larsson M, Alm E, Yekta SS, Svensson BH, et al. 454 pyrosequencing analyses of bacterial and archaeal richness in 21 full-scale biogas digesters. *FEMS Microbiol Ecol* 2013; **85**: 612–626.

14. Glöckner FO, Yilmaz P, Quast C, Gerken J, Beccati A, Ciuprina A, et al. 25 years of serving the community with ribosomal RNA gene reference databases and tools. *J Biotechnol* 2017; **261**: 169–176.

15. Quast C, Pruesse E, Yilmaz P, Gerken J, Schweer T, Yarza P, et al. The SILVA ribosomal RNA gene database project: improved data processing and web-based tools. *Nucleic Acids Res* 2013; **41**: 590–596.

16. Konstantinidis KT, Tiedje JM. Towards a genome-based taxonomy for prokaryotes. *J Bacteriol* 2005; **187**: 6258–6264.

17. Feike J, Jürgens K, Hollibaugh JT, Krüger S, Jost G, Labrenz M. Measuring unbiased metatranscriptomics in suboxic waters of the central Baltic Sea using a new in situ fixation system. *ISME J* 2012; **6**: 461–470.

18. Weinbauer MG, Fritz I, Wenderoth DF, Hofle MG. Simultaneous extraction from bacterioplankton of total RNA and DNA suitable for quantitative structure and function analyses. *Appl Environ Microbiol* 2002; **68**: 1082–1087.

19. Satinsky BM, Gifford SM, Crump BC, Moran MA. Use of internal standards for quantitative metatranscriptome and metagenome analysis. *Methods Enzymol* 2013; **531**: 237–250.

20. Peng Y, Leung HCM, Yiu SM, Chin FYL. IDBA-UD: A de novo assembler for single-cell and metagenomic sequencing data with highly uneven depth. *Bioinformatics* 2012; **28**: 1420–1428.

21. Kopylova E, Noé L, Touzet H. SortMeRNA: Fast and accurate filtering of ribosomal RNAs in metatranscriptomic data. *Bioinformatics* 2012; **28**: 3211–3217.

22. Kiełbasa SM, Wan R, Sato K, Horton P, Frith MC. Adaptive seeds tame genomic sequence comparison. *Genome Res* 2011; **21**: 487–493.

23. Hyatt D, Chen G-L, LoCascio PF, Land ML, Larimer FW, Hauser LJ. Prodigal: prokaryotic gene recognition and translation initiation site identification. *BMC Bioinformatics* 2010; **11**: 119.

24. Rasko DA, Myers GSA, Ravel J. Visualization of comparative genomic analyses by BLAST score ratio. *BMC Bioinformatics* 2005; **6**: 1–7.

25. Blum M, Chang HY, Chuguransky S, Grego T, Kandasaamy S, Mitchell A, et al. The InterPro protein families and domains database: 20 years on. *Nucleic Acids Res* 2021; **49**: D344–D354.

26. Huerta-Cepas J, Forslund K, Coelho LP, Szklarczyk D, Jensen LJ, Von Mering C, et al. Fast genome-wide functional annotation through orthology assignment by eggNOG-mapper. *Mol Biol Evol* 2017; **34**: 2115–2122.

27. Chevreux B, Wetter T, Suhai S, others. Genome sequence assembly using trace signals and additional sequence information. *Ger. Conf. Bioinforma.* 1999. pp 45–56.

28. Joshi NA, Fass J, others. Sickle: A sliding-window, adaptive, quality-based trimming tool for FastQ files (Version 1.33)[Software]. 2011.

29. Langmead B, Trapnell C, Pop M, Salzberg SL. Ultrafast and memory-efficient alignment of short DNA sequences to the human genome. *Genome Biol* 2009; **10**.

30. Liao Y, Smyth GK, Shi W. featureCounts: an efficient general purpose program for assigning sequence reads to genomic features. *Bioinformatics* 2014; **30**: 923–930.

31. Buchfink B, Reuter K, Drost H-G. Sensitive protein alignments at tree-of-life scale using DIAMOND. *Nat Methods* 2021; **18**: 366–368.

32. Von Meijenfeldt FAB, Arkhipova K, Cambuy DD, Coutinho FH, Dutilh BE. Robust taxonomic classification of uncharted microbial sequences and bins with CAT and BAT. *Genome Biol* 2019; **20**: 1–14.

33. Lahme S, Callbeck CM, Eland LE, Wipat A, Enning D, Head IM, et al. Comparison of sulfide-oxidizing *Sulfurimonas* strains reveals a new mode of thiosulfate formation in subsurface environments. *Environ Microbiol* 2020; **22**: 1784–1800.

34. Grote J, Schott T, Bruckner CG, Glockner FO, Jost G, Teeling H, et al. Genome and physiology of a model Epsilonproteobacterium responsible for sulfide detoxification in marine oxygen depletion zones. *Proc Natl Acad Sci U S A* 2012; **109**: 506–510.

35. Sievert SM, Scott KM, Klotz MG, Chain PSG, Hauser LJ, Hemp J, et al. Genome of the Epsilonproteobacterial chemolithoautotroph *Sulfurimonas denitrificans*. *Appl Environ Microbiol* 2008; **74**: 1145–1156.

36. Henkel J V, Vogts A, Werner J, Neu TR, Spröer C, Bunk B, et al. *Candidatus* Sulfurimonas marisnigri sp. nov. and *Candidatus* Sulfurimonas baltica sp. nov., thiotrophic manganese oxide reducing chemolithoautotrophs of the class *Campylobacteria* isolated from the pelagic redoxclines of the Black Sea an. *Syst Appl Microbiol* 2021; **44**.

37. Henkel J V, Dellwig O, Pollehne F, Herlemann DPR, Leipe T, Schulz-Vogt HN. A bacterial isolate from the Black Sea oxidizes sulfide with manganese(IV) oxide. *Proc Natl Acad Sci U S A* 2019; **116**: 12153–12155.

38. Siu T, Jia CQ. Kinetics of reaction of sulfide with thiosulfate in aqueous solution. *Ind Eng Chem Res* 1999; **38**: 1306–1309.

39. Yao W, Millero FJ. The rate of sulfide oxidation by δMnO_2_ in seawater. *Geochim Cosmochim Acta* 1993; **57**: 3359–3365.

40. Cline JD. Spectrophotometric determination of hydrogen sulfide in natural waters. *Limnol Oceanogr* 1969; **14**: 454–458.

41. Schulz HD. Conceptual models and computer models. In: Schulz HD, Zabel M (eds). *Marine Geochemistry*. 2006. Springer, pp 513–547.

42. Gregg MC, Yakushev E. Surface ventilation of the Black Sea’s cold intermediate layer in the middle of the western gyre. *Geophys Res Lett* 2005; **32**: 1–4.

43. Diepenbroek M, Glöckner FO, Grobe P, Güntsch A, Huber R, König-Ries B, et al. Towards an integrated biodiversity and ecological research data management and archiving platform: the German federation for the curation of biological data (GFBio). In: Plödereder E, Grunske L, Schneider E, Ull D (eds). *Inform. 2014*. 2014. Gesellschaft für Informatik e.V., Bonn, pp 1711–1721.

44. Yilmaz P, Kottmann R, Field D, Knight R, Cole JR, Amaral-Zettler L, et al. Minimum information about a marker gene sequence (MIMARKS) and minimum information about any (x) sequence (MIxS) specifications. *Nat Biotechnol* 2011; **29**: 415–420.
