# Supplementary material for: Biological manganese-dependent sulfide oxidation impacts elemental gradients in redox-stratified systems: indications from the Black Sea water column
Source: ISME J. 2022 Feb 5;16(6):1523–33. doi: 10.1038/s41396-022-01200-3 (PMC9122950; doi:10.1038/s41396-022-01200-3)
Supplement: Supplementary file 2 — Supplementary tables S1–S4 [file 41396_2022_1200_MOESM2_ESM.docx]

Table S1: Relative 16S rRNA gene abundance of *Campylobacterota* annotated taxonomic groups as percentages of total bacterial 16S rRNA gene reads.

| CTD cast | Depth (m) | *Sulfurimonas* spp. | *Arcobacteraceae* | others |
| --- | --- | --- | --- | --- |
| P0014F08 | 82.3 | 0.15 | 0.06 | 0.00 |
|  | 88.8 | 1.01 | 0.22 | 0.00 |
|  | 95.5 | 4.89 | 0.45 | 0.01 |
|  | 100.4 | 6.07 | 0.23 | 0.00 |
|  | 105.0 | 11.25 | 0.37 | 0.01 |
|  | 109.4 | 14.06 | 0.46 | 0.02 |
|  |  |  |  |  |
| P0014F10 | 82.3 | 0.09 | 0.03 | 0.00 |
|  | 85.3 | 0.12 | 0.04 | 0.00 |
|  | 89.5 | 1.32 | 0.22 | 0.00 |
|  | 92.5 | 4.40 | 0.55 | 0.00 |
|  | 102.5 | 12.01 | 0.41 | 0.02 |
|  | 109.4 | 9.92 | 0.15 | 0.01 |
|  |  |  |  |  |
| V0014F13 | 88.1 | 0.36 | 0.06 | 0.00 |
|  | 98.4 | 9.02 | 0.59 | 0.01 |
|  | 101.1 | 7.68 | 0.19 | 0.01 |
|  | 109.8 | 12.95 | 0.13 | 0.01 |

Table S2: Relative 16S rRNA abundance of *Campylobacterota* annotated taxonomic groups as percentages of total bacterial 16S rRNA reads.

| CTD cast | Depth (m) | *Sulfurimonas* spp. | *Arcobacteraceae* | others |
| --- | --- | --- | --- | --- |
| P0014F08 | 82.3 | 2.15 | 0.02 | 0.01 |
|  | 88.8 | 8.84 | 0.32 | 0.01 |
|  | 95.5 | 25.04 | 0.69 | 0.03 |
|  | 100.4 | 24.10 | 0.31 | 0.03 |
|  | 105.0 | 32.75 | 0.43 | 0.01 |
|  | 109.4 | 33.36 | 0.50 | 0.02 |
|  |  |  |  |  |
| P0014F10 | 82.3 | 1.92 | 0.09 | 0.01 |
|  | 85.3 | 2.22 | 0.12 | 0.01 |
|  | 89.5 | 7.42 | 0.17 | 0.01 |
|  | 92.5 | 20.28 | 0.59 | 0.02 |
|  | 102.5 | 37.48 | 0.68 | 0.04 |
|  | 109.4 | 25.86 | 0.19 | 0.02 |
|  |  |  |  |  |
| V0014F13 | 88.1 | 3.69 | 0.13 | 0.01 |
|  | 98.4 | 29.87 | 0.61 | 0.03 |
|  | 101.1 | 20.87 | 0.16 | 0.02 |
|  | 109.8 | 35.42 | 0.27 | 0.02 |

Table S3: Relative 16S rRNA gene abundance of *Campylobacterota* annotated taxonomic groups as percentages of total *Campylobacterota* 16S rRNA gene reads.

| CTD cast | Depth (m) | *Sulfurimonas* spp. | *Arcobacteraceae* | others |
| --- | --- | --- | --- | --- |
| P0014F08 | 82.3 | 69.91 | 29.17 | 0.92 |
|  | 88.8 | 82.01 | 17.90 | 0.09 |
|  | 95.5 | 91.39 | 8.47 | 0.14 |
|  | 100.4 | 96.39 | 3.58 | 0.03 |
|  | 105.0 | 96.76 | 3.16 | 0.08 |
|  | 109.4 | 96.71 | 3.14 | 0.16 |
|  |  |  |  |  |
| P0014F10 | 82.3 | 78.43 | 21.57 | 0.00 |
|  | 85.3 | 74.44 | 25.56 | 0.00 |
|  | 89.5 | 85.22 | 14.47 | 0.31 |
|  | 92.5 | 88.84 | 11.11 | 0.05 |
|  | 102.5 | 96.54 | 3.28 | 0.18 |
|  | 109.4 | 98.40 | 1.45 | 0.15 |
|  |  |  |  |  |
| V0014F13 | 88.1 | 85.02 | 14.29 | 0.70 |
|  | 98.4 | 93.79 | 6.12 | 0.09 |
|  | 101.1 | 97.55 | 2.37 | 0.08 |
|  | 109.8 | 98.93 | 0.98 | 0.09 |

Table S4: Relative 16S rRNA abundance of *Campylobacterota* annotated taxonomic groups as percentages of total *Campylobacterota* 16S rRNA reads.

| CTD cast | Depth (m) | *Sulfurimonas* spp. | *Arcobacteraceae* | others |
| --- | --- | --- | --- | --- |
| P0014F08 | 82.3 | 98.19 | 1.13 | 0.68 |
|  | 88.8 | 96.40 | 3.49 | 0.11 |
|  | 95.5 | 97.21 | 2.68 | 0.11 |
|  | 100.4 | 98.62 | 1.28 | 0.10 |
|  | 105.0 | 98.69 | 1.28 | 0.03 |
|  | 109.4 | 98.46 | 1.49 | 0.05 |
|  |  |  |  |  |
| P0014F10 | 82.3 | 95.21 | 4.43 | 0.36 |
|  | 85.3 | 94.41 | 5.21 | 0.39 |
|  | 89.5 | 97.58 | 2.27 | 0.16 |
|  | 92.5 | 97.06 | 2.82 | 0.12 |
|  | 102.5 | 98.13 | 1.78 | 0.10 |
|  | 109.4 | 99.17 | 0.74 | 0.08 |
|  |  |  |  |  |
| V0014F13 | 88.1 | 96.29 | 3.42 | 0.29 |
|  | 98.4 | 97.92 | 1.98 | 0.10 |
|  | 101.1 | 99.17 | 0.76 | 0.07 |
|  | 109.8 | 99.20 | 0.75 | 0.06 |
